# Supplementary material for: The interactome of intact mitochondria by cross-linking mass spectrometry provides evidence for coexisting respiratory supercomplexes
Source: Mol Cell Proteomics. 2017 Dec 8;17(2):216–32. doi: 10.1074/mcp.RA117.000470 (PMC5795388; doi:10.1074/mcp.RA117.000470)
Supplement: Supplemental Data [file supp_RA117.000470_133922_0_supp_23978_fzffwf.zip › spectra_annotation/mito_spectra_annotation/92-1-2-1-8-1.pdf]

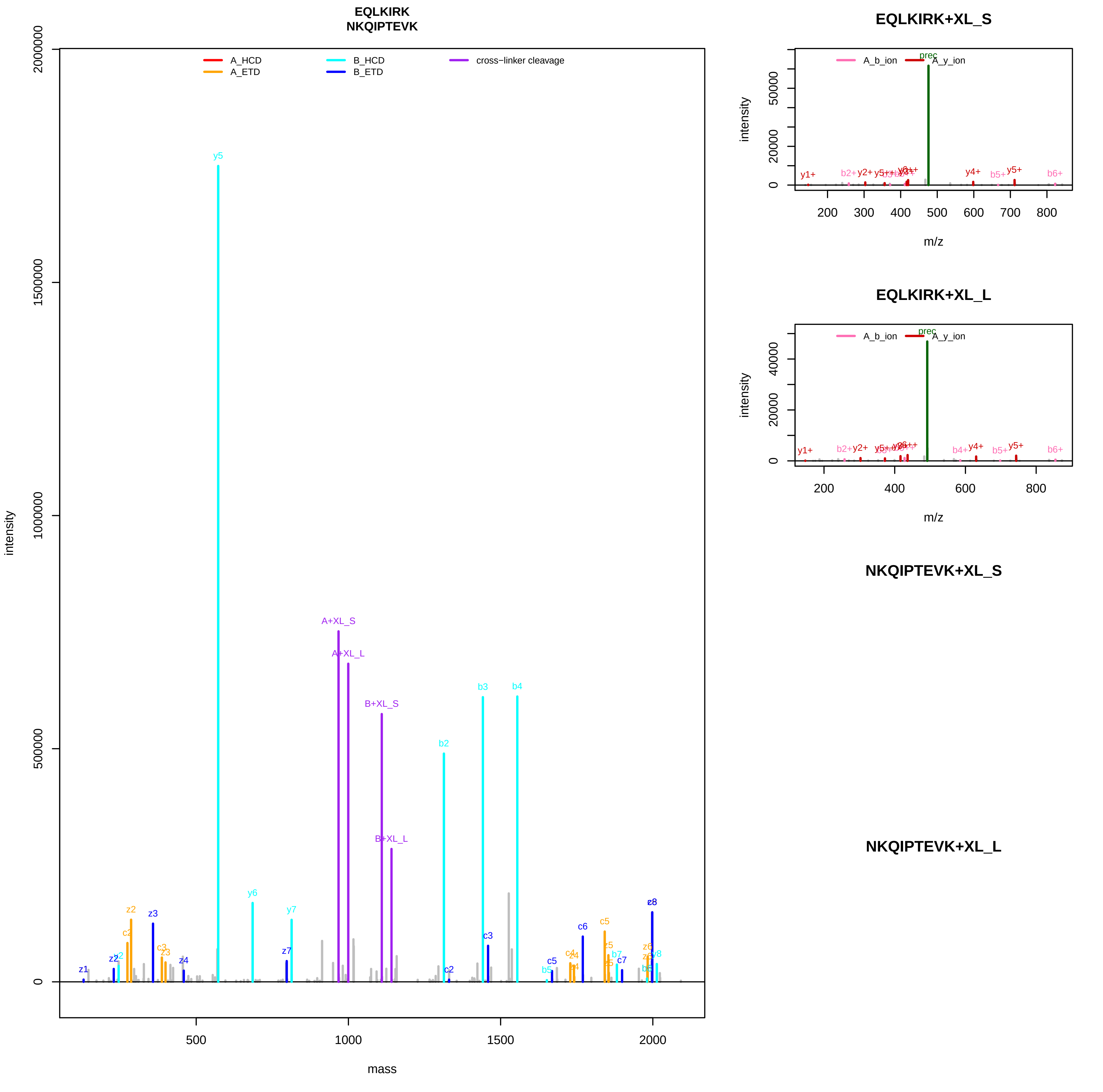
EQLKIRK
NKQIPTEVK
A_HCD
A_ETD
B_HCD
B_ETD
cross−linker cleavage
intensity
mass
0
500000
1000000
1500000
2000000
500
1000
1500
2000
y5
A+XL_S
A+XL_L
B+XL_S
B+XL_L
b2
b3
b4
y6
y7
z1
z2
z3
z4
z7
c2
c3
c5
c6
c7
z8
b5
b7
y8
EQLKIRK+XL_S
EQLKIRK+XL_L
NKQIPTEVK+XL_S
NKQIPTEVK+XL_L
A_b_ion
A_y_ion
prec
m/z
y1+
b2+
y2+
y4+
b5+
y5+
b6+
b4+
